# Transcriptome and metabolite analysis reveal the drought tolerance of foxtail millet significantly correlated with phenylpropanoids-related pathways during germination process under PEG stress

**DOI:** 10.1186/s12870-020-02483-4

**Published:** 2020-06-15

**Authors:** Aili Yu, Jinfeng Zhao, Zhenhua Wang, Kai Cheng, Peng Zhang, Gang Tian, Xin Liu, Erhu Guo, Yanwei Du, Yuwen Wang

**Affiliations:** Millet Research Institute, Shanxi Academy of Agricultural Sciences, Changzhi, 046011 China

**Keywords:** Foxtail millet, Germination, Transcriptome analysis, Phenylpropanoids-related pathways and metabolites, PEG stress

## Abstract

**Background:**

Foxtail millet [*Setaria italica* (L.) P. Beauv.] is an excellent crop known for its superior level of drought tolerance across the world. Especially, less water is needed during its germination period than the other cereal crops. However, the knowledge of the mechanisms underlying the abiotic stress effects on seed germination of foxtail millet is largely unknown.

**Results:**

The water uptake pattern of foxtail millet seeds was ploted during germination period, according to which the germination time course of millet was separated into three phases. We sequenced the transcriptome of foxtail millet seeds, which were treated by PEG during different germination phases after sowing. The transcriptional studies revealed that more DEGs were identified during the further increase in water uptake period (phase III) than during the rapid initial uptake period (phase I) and the plateau period (phase II) under PEG stress. The pathway analysis of DEGs showed that the highly enriched categories were related to phenylpropanoid biosynthesis, plant hormone signal transduction and phenylalanine metabolism during phase III. The 20 phenylpropanoids-related genes of germinating foxtail millet were found to be down-regulated during the further increase in water uptake period under PEG stress. Further expression analysis identified 4 genes of phenylalanine ammonia-lyase, 4-coumarate-CoA ligase 3, cinnamoyl-CoA reductase 1, cationic peroxidase SPC4 in phenylpropanoids-related pathway, which played important roles in foxtail millet in response to PEG stress during different germination periods. The studies of metabolites in phenylpropanoid biosynthesis pathway revealed that higher amount of cinnamic acid was accumulated in germinating seeds under PEG stress, while the contents of p-coumaric acid, caffeic acid, ferulic acid and sinapic acid were decreased. And the effects of five phenolic compounds on germination and growth of foxtail millet showed that 1 mM concentration of cinnamic acid inhibited shoot and root growth, especially root development. Ferulic acid, caffeic acid, sinapic acid and p-coumaric acid could increase the root length and root/sprout in lower concentration.

**Conclusions:**

These findings suggest that key genes and metabolites of foxtail millet related with phenylpropanoids pathway may play prominent roles in the regulation of resistance to drought during germination. Foxtail millet can probably avoid drought by regulating the levels of endogenous allelochemicals.

## Background

Drought is one of the most frequent and severe abiotic stress factors, which adversely affects plant growth and crop productivity in many arid and semiarid regions [1–3]. Additionally, seasonal droughts often occur unevenly in the non-arid regions [2]. In particular, droughts in spring severely impact the germination of seeds.

Seed germination commences with the uptake of water by the dry seed (imbibition), and is completed when usually the radicle extends to penetrate the structures that surround it [4]. Uptake of water by a mature dry seed is triphasic, including a rapid initial uptake (phase I), a followed plateau phase (phase II), and a further increase in water uptake (phase III). Phase III occurs only after germination is completed, while the embryonic axes elongate and the radicles protrude, often called visible germination [5]. During germination process, the influx of water into the cells of dry seeds results in rapidly resuming metabolic activity of the quiescent dry seed, and a series of complex structures, as well as physiological and molecular changes occur, such as temporary membrane structural perturbations, mobilization of the major storage reserves, recruitment of polysomes, the translation of preformed mRNAs, mRNA de novo synthesis [6, 7]. All the cellular and metabolic events occur in the nondormant seeds (before the completion of germination) and the imbibed dormant seeds, but the metabolic activities of the latter are subtly different from those of the former [5]. The imbibed mature seed is sensitive to different environmental factors during germination process. However, the knowledge about the physiological and molecular mechanisms underlying the environmental effects on germination was largely lacking [8, 9].

Foxtail millet [*Setaria italica* (L.) P. Beauv.] is one of the oldest cereals and is thought to have played an important role in ancient civilization as a staple crop [10]. Foxtail millet is known as a relatively drought-tolerant crop across the world, and grows in arid and semi-arid regions. Its morphological and anatomical characteristics give it strong drought resistance, such as thick cell wall, dense reticulate root system and small leaf area [11]. Foxtail millet not only has strong drought tolerance, but also possesses small diploid genome sequence (about 515 Mb), strong inbreeding, short growth period and abundant germplasm resources. These features make millet an ideal model system for studying abiotic challenges [12, 13]. Moreover, it was reported that foxtail millet had significantly high water use efficiency compared with wheat, maize and sorghum [11]. Especially, it needs less water in germination period than the other cereal crops, which can germinate when water absorption accounts for about 26% of seed weight [14]. However, the metabolite changes and transcriptome reprogramming of foxtail millet in response to drought stress during germination process are rarely studied.

Phenylpropanoids, which are plant-specific natural products, play important functions during growth, development, and environmental interactions [15, 16]. Phenylpropanoids are precursors of a wide range of phenolic compounds, such as flavonoids, isoflavonoids, anthocyanins, plant hormones, phytoalexins, and lignins [17, 18]. Phenylpropanoids are synthesized from phenylalanine via the central phenylpropanoid pathway. The emergence of the phenylpropanoid pathway in plants is an important evolutionary adaptation that enables plant defense against abiotic and biotic stresses [19]. Phenylpropanoids are in response to environmental cues and serve important functions in several different pathways including plant defense against pathogens and predators, protection from UV irradiation, signal transduction and communication with other organisms, and regulatory molecules [15, 17, 19–22]. And many phenylpropanoids and related metabolites have been reported to be allelochemicals, such as cinnamic acid and its hydroxylated derivatives, which can prohibit the germination of many plant seeds [23]. However, the function of phenylpropanoids in seed germination of foxtail millet is still unclear.

RNA-Seq is an important technology that has been used to elucidate the complexity of regulation of gene expression during various stress conditions, as well as to obtain genome-wide estimations of relative gene expression. Rahman et al. sequenced the salinity responsive leaf transcriptome of the susceptible and tolerant finger millet. They found in the tolerant Trichy 1, the genes of several functional groups, such as transporters and transcription factors, were highly up-regulated, and genes involved in flavonoid biosynthesis were down-regulated specifically. Salinity stress inhibited photosynthesis related genes in the susceptible genotype [24]. But the transcriptional profile analysis of the germinating seeds remains limited in foxtail millet under drought stress.

In this study, we aimed to understand the transcriptional and metabolic basis of drought responsiveness in foxtail millet during germination period. We plotted the figure associated with water uptake pattern of germinating seeds. The transcriptome analysis using RNA-Seq was performed for germination seeds in response to osmotic stress induced by polyethylene glycol (PEG). Many differentially expressed genes (DEGs) were identified separately in different phases of seed germination. Phenylpropanoid biosynthesis was found to be the highly enriched category. The related DEGs and metabolites of phenylpropanoid pathway were further analyzed in foxtail millet under PEG stress during different growth stages. These results provide an opportunity to elucidate the molecular mechanism underlying drought resistance of foxtail millet during germination process.

## Results

### Phenotypic symptoms of foxtail millet during germination period under PEG stress

Total 8 foxtail millet cultivars were screened for their drought tolerances by germination percentage, relative sprout length and relative root length. Among them, cultivar Jingu 20 showed the highest germination percentage and relative root length, indicating its strongest dehydration tolerance (Shown in Additional file 1: Figure S1 and Additional file 2: Figure S2). Thus Jingu 20 was selected to understand the phenotypic and molecular basis of drought tolerance mechanisms.

**Fig. 1:**
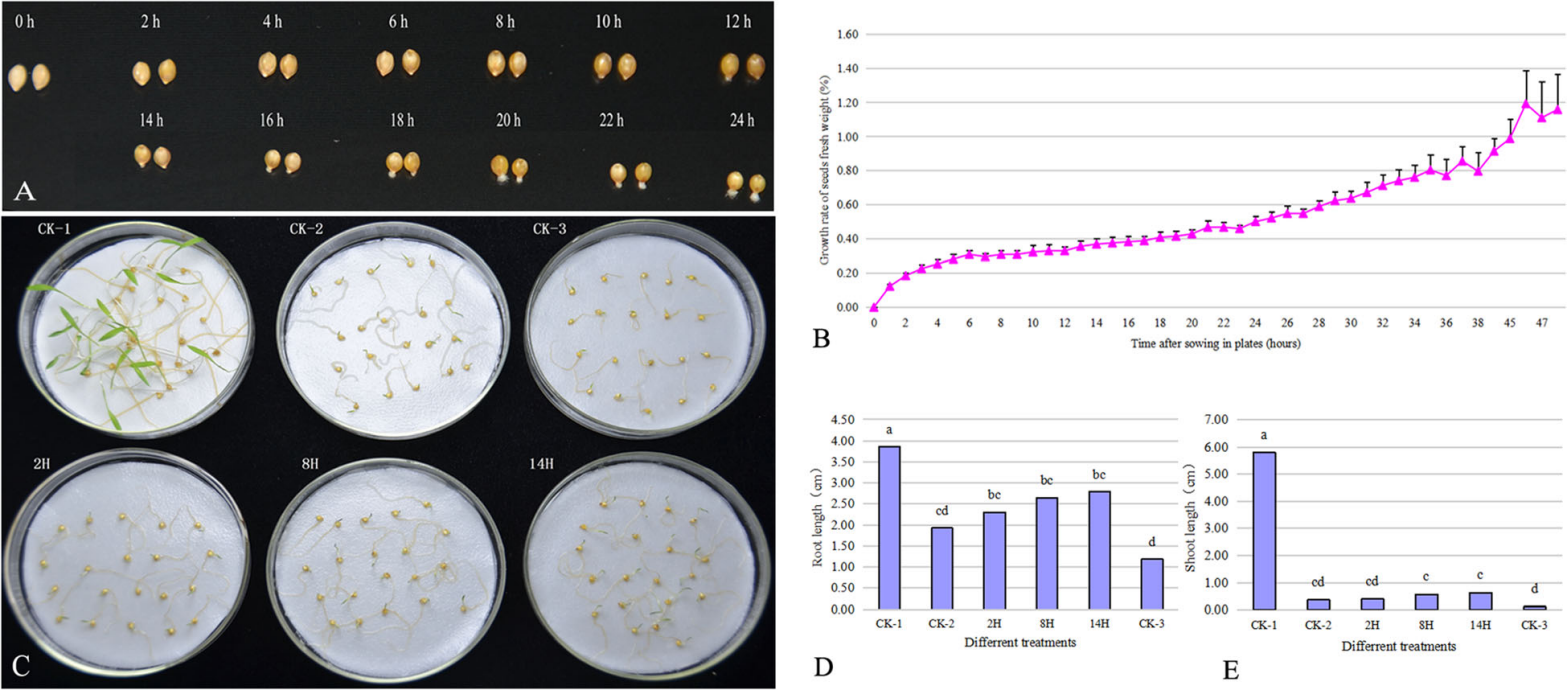
The phenotypic symptoms of foxtail millet during germination period under normal and PEG stress conditions. **a** The phenotypic symptoms of foxtail millet seeds at 0 h, 2 h, 4 h, 6 h, 8 h, 10 h, 12 h, 14 h, 16 h, 18 h, 20 h, 22 h, 24 h after sowing under normal conditions. **b** Water uptaking pattern of foxtail millet seeds during germination period under normal conditions. **c** Differential responses of foxtail millet treated by PEG at different timepoints after sowing. **d** Root length of foxtail millet treated by PEG at different timepoints after sowing. **e**. Shoot length of foxtail millet treated by PEG at different timepoints after sowing. **CK-1**, foxtail millet growing under normal condition. **CK-2**, foxtail millet sowed under PEG stress condition. **CK-3**, foxtail millet treated by PEG after absorbing water for 40 min. **2H**, foxtail millet treated by PEG after sowing for 2 h under normal conditions. **8H**, foxtail millet treated by PEG after sowing for 8 h under normal conditions. **14H**, foxtail millet treated by PEG after sowing for 14 h under normal conditions. The mean values and SD in (**d**) and (**e**) were calculated using one-way ANOVA followed by Tukey HSD multiple comparison

The water uptake pattern and germination phenotype of Jingu 20 seeds were analyzed under 24 °C ~ 26 °C conditions in our culture room (Fig. 1). The pattern included three stages of a rapid initial uptake, a followed plateau phase and a further increase in water uptake (Fig. 1b), which were consistent with the previous results reported by Bewley [5]. The rapid initial uptake period (phase I) was from 0 h to 6 h. The plateau phase (phase II) was from 6 h to 12 h. A further increase in water uptake (phase III) commenced at 12 h after sowing, at which the radicles of few seeds protruded. After sowing for 14 h, the radicles of all seeds penetrated the structures that surrounded them (Fig. 1a).

The phenotypic symptoms of Jingu 20 treated by PEG at 2 h (2H), 8 h (8H) and 14 h (14H) after sowing under water condition were shown in Fig. 1c. Using seeds grown at water condition (CK-1) as controls, PEG leaded to remarkable reduction in the growth of roots and shoots of 2H, 8H, 14H, CK-2 (seeds sowed under PEG stress) and CK-3 (seeds treated by PEG after absorbing water for 40 min). The length of roots and shoots were found to be affected more severely in CK-3 than in 2H, 8H, 14H. No significant differences were found in the length of the growth zone between of 2H, 8H, 14H and CK-2. And PEG stress slightly affected the growth of roots (Fig. 1d), but significantly reduced the length of shoot (Fig. 1e). These results indicated that the shoots suffered more inhibition than the roots in foxtail millet under PEG stress during germination.

### Transcriptome analysis of foxtail millet in response to drought stress during seed germination

#### Drought responsive transcriptome profiling

To gain a global view on the drought induced changes at transcriptome level and drought responsive metabolic pathways in foxtail millet during germination, twelve cDNA libraries of Jingu 20 were sequenced from CK2H (collected during phase I, without PEG stress treatment), CK8H (collected during phase II, without PEG stress treatment)), CK14H (collected during phase III, without PEG stress treatment), P2H (collected during phase I, treated by PEG stress for 1 h and 3 h), P8H (collected during phase II, treated by PEG stress for 1 h and 3 h), P14H (collected during phase III, treated by PEG stress for 1 h and 3 h). After removing low quality reads and adapter sequences, a total of 38,845,760 and 39,679,812 (CK2H), 35,384,758 and 36,744,034 (P2H), 56,164,700 and 43,525,742 (CK8H), 52,411,398 and 47,479,886 (P8H), 46,838,670 and 49,151,796 (CK14H), 41,676,048 and 48,986,294 (P14H) clean reads were generated, which were listed in Additional file 8: Table S1. These libraries with Q30 > 85.02% were perfectly matched to the foxtail millet reference sequences from 78.50 to 82.84%.

#### Identification and functional annotation of differentially expressed genes

Differentially expressed genes (DEGs) during the progression of stresses show the stresses responsiveness and their putative roles in drought tolerance. DEGs were identified at a absolute threshold of fold change ≥2 and FDR ≤0.01 (FDR, false discovery rate). In phase I of foxtail millet germination, total 6 genes were detected in PEG stress samples (P2H) with CK2H (without PEG stress) as controls, of which 3 were up-regulated and 3 down-regulated (Additional file 3: Figure S3 and Table S3). At phase II, 42 genes were differentially regulated in the PEG stress samples (P8H) in comparison with CK8H (without PEG stress), including 24 up-regulated and 18 down-regulated genes. During phase III, a total of 302 up-regulated and 355 down-regulated genes were identified in PEG treated samples (P14H) when compared with the controls (CK14H, without PEG stress). The result showed that more DEGs were found during the further increase in water uptake period under drought stress.

COG refers to clusters of orthologous groups for eukaryotes. In this database, every protein is assumed to be evolved from a common ancestor protein. According to COG, 228 DEGs were classified into 21 different COG categories in the comparison of CK14H vs. P14H during phase III. Among these categories, the “general function prediction only”, “signal transduction mechanisms”and “transcription” were found to be the largest group in the compared groups (Additional file 4: Figure S4).

**Fig. 2:**
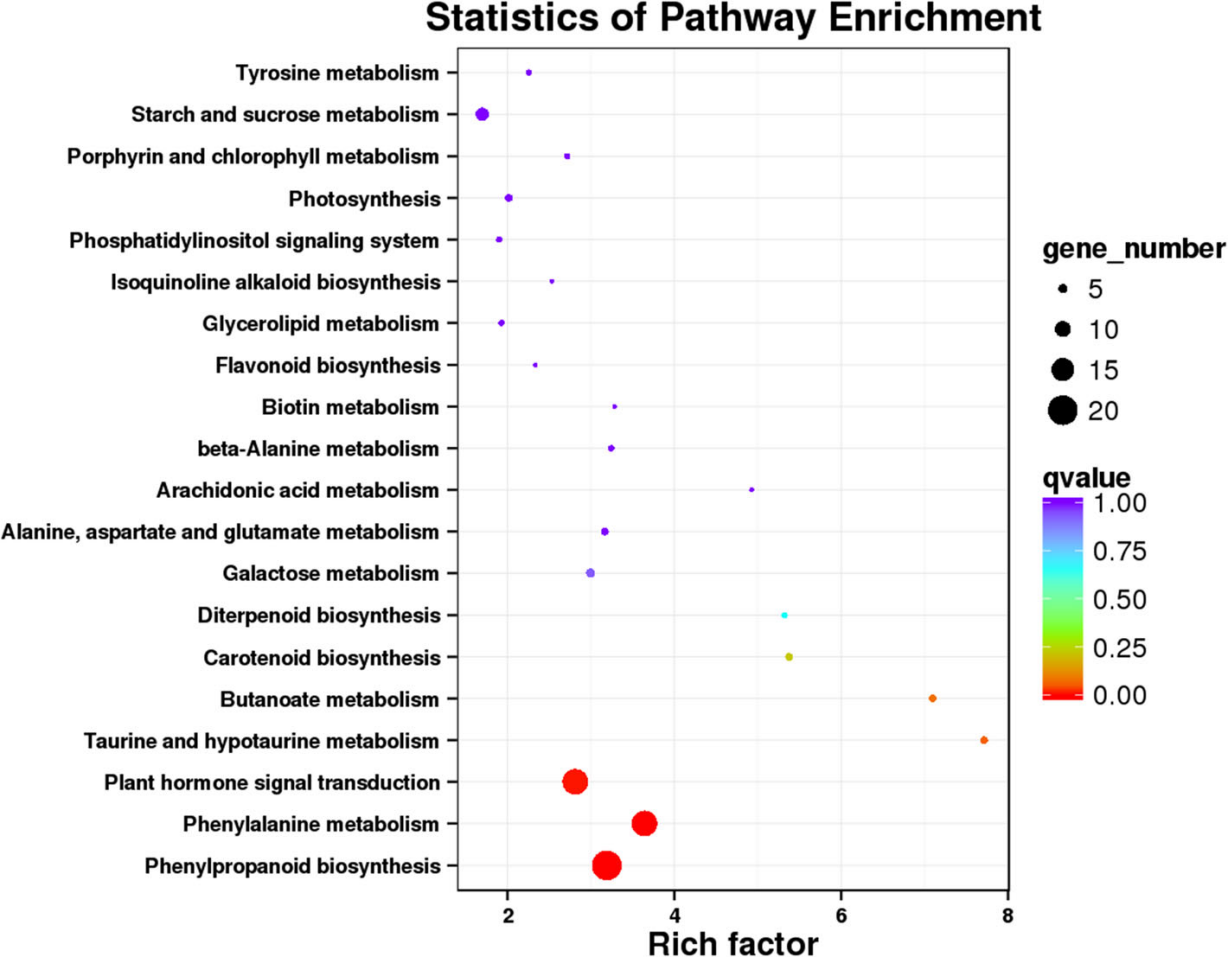
The pathways with the most significant Q value in CK14H vs. P14H. The x-axis indicated rich factor of DEGs belong to the corresponding pathway. The left y-axis represented the pathways. The sizes of bubble represented the number of DEGs in the corresponding pathway, and the colors of the bubble represented the enrichment Q value of the corresponding pathway

To further understand the biological functions of genes under drought stress, all DGEs were analyzed against the kyoto encyclopedia of genes and genomes (KEGG). In CK14H vs. P14H of phase III, 121 DEGs were mapped to the reference canonical pathways (Additional file: Table S3), which were classified into 70 functional categories (Additional file: Figure S5). Eight DEGs were found in CK8H vs. P8H, including 7 KEGG functional categories, and one DEG in CK2H vs. P2H (Additional file: Table S3). Using KEGG, the significantly enriched categories were identified, such as “phenylpropanoid biosynthesis, plant hormone signal transduction and phenylalanine metabolism” in CK14H vs. P14H (Fig. 2). Phenylpropanoid biosynthesis was dramatically enriched in CK8H vs. P8H (Additional file: Figure S6). The enrichment of other secondary metabolic pathways during different time point was not significant (Fig. 2 and Additional file: Figure S6 and Table S3). These findings suggested that many phenylpropanoids-related genes might play important roles in drought response during the further increase in water uptake period. Therefore, the following analysis mainly focused on phase III of foxtail millet germination under PEG stress.

#### Drought response transpoters in foxtail millet

Multiple transporters play important roles in plants in response to various environmental stimuli [25]. In this study, 37 genes encoding transporters were identified in CK14H vs. P14H of Jingu 20 under PEG stress, and 1 gene in CK8H vs. P8H (Additional file: Table S4). Two outer envelope pore proteins and 2 S-adenosylmethionine carriers were up-regulated in CK14H vs. P14H. The expression levels of aquaporin PIP2–5, cation/calcium exchanger 1, glycerol-3-phosphate transporter 4, bidirectional sugar transporter SWEET1b and mannan endo-1,4-beta-mannosidase 1 were separately increased in the same sample. Amino-acid permease BAT1 homolog was found to be up-regulated in CK14H vs. P14H and CK8H vs. P8H. Cation/calcium exchanger NIP2–2 was down-regulated in CK14H vs. P14H under PEG stress.

#### Drought response signal transduction elements in foxtail millet

Protein kinases and phosphatases turn on or off stress responses by phosphorylation/ dephosphorylation to activate transcription factors and other genes related to stress tolerance [26, 27]. Total 38 protein kinases were found to be differentially regulated in CK14H vs. P14H (Additional file: Table S4). Among them, 6 genes were up-regulated, which encoded CBL-interacting protein kinase 16, SNF1-related protein kinase regulatory subunit gamma-like PV42a, SNF1-related protein kinase regulatory subunit gamma-1, two proline-rich receptor-like protein kinase PERK2, proline-rich receptor-like protein kinase PERK9. Out of 12 differentially expressed phosphatases, nine phosphatases 2C were found to be up-regulated in CK14H vs. P14H. The transcript of one phosphatase 2C was increased by PEG stress in CK8H vs. P8H of foxtail millet. Furthermore, 2 abscisic acid receptor PYL4 and 2 JAZ-like repressors of jasmonate signaling (Protein TIFY 10B and CASP-like protein 4A2) were found to be down-regulated under PEG stress in CK14H vs. P14H.

#### Drought response transcription factors in foxtail millet

Transcription factors regulate the expression of many downstream genes at the transcriptional level and control many biological processes such as cell division, growth, and response to environmental stress [28]. In this study, 56 genes encoding transcription factors were identified in CK14H vs. P14H of Jingu 20 under PEG stress (Additional file: Table S4). These genes mainly included 14 ethylene-responsive transcription factor, 7 MYB, 6 heat stress transcription factor, 4 bZIP transcription factors, 3 zinc finger CCCH domain-containing protein, 3 WRKY, 3 transcription factor bHLH, 2 dehydration-responsive element-binding protein, 1 AP2/ERF and B3 domain-containing protein. The transcripts of 6 ethylene-responsive transcription factor genes were up-regulated, and 8 ones were down-regulated. Four bZIP transcription factors and three zinc finger CCCH domain-containing protein were found to be up-regulated. The levels of 4 heat stress transcription factor, 2 MYB, 1 WRKY and 1 dehydration-responsive element-binding protein also increased in CK14H vs. P14H. Three ethylene-responsive transcription factor and 1 homeobox-leucine zipper protein were identified in CK8H vs. P8H. Activation of ethylene-responsive transcription factor, heat stress transcription factor, MYB, WRKY, etc. showed that foxtail millet modulated stress tolerance during germination period under PEG conditions.

#### Drought responsiveness of genes related to phytohormone biosynthesis in foxtail millet

Phytohormones play important roles in response and adaptation to stress by reducing or mitigating the negative effects of stress [24]. In CK14H vs. P14H, genes involved in auxin, cytokinine, ethylene, salicylic acid biosynthesis were found to be down-regulated (Additional file: Table S4). The expression levels of three 9-cis-epoxycarotenoid dioxygenase 1 and beta-carotene 3-hydroxylase related to abscisic acid biosynthesis were increased under PEG stress. Three genes encoding for gibberellin 2-beta-dioxygenase 1, ent-copalyl diphosphate synthase 1 and cytochrome P450 88A1 related to gibberellin biosynthesis were promoted by PEG stress. One gene of phenylalanine ammonia-lyase involved in salicylic acid biosynthesis was depressed by PEG treatment.

#### Regulation of genes involved in osmotic homeostasis under PEG stress

Plants often overcome the adverse effects of osmotic stress by accumulating metabolites or compatible solutes. Late embryogenesis abundant protein and dehydrin associated with tolerance against water stress [24]. In CK14H vs. P14H, 9 genes encoding late embryogenesis abundant protein and 1 dehydrin were found to be up-regulated (Additional file: Table S4). Phosphoethanolamine N-methyltransferase involved in the biosynthesis of choline was up-regulated, which is the upstream gene of glycine betaine production. The transcript of phosphoethanolamine N-methyltransferase was increased in CK8H vs. P8H. Accumulation of proline is the adaptive response of plants against environmental stresses [29]. One up-regulated gene of gamma-glutamyl phosphate reductase and one down-regulated gene of proline dehydrogenase 2 were found in CK14H vs. P14H, which might lead to the accumulation of proline. Sucrose and raffinose acts as compatible osmolytes in response to stress [30, 31]. In CK14H vs. P14H, one gene encoding alpha-galactosidase involved in production of galactose, glucose, sucrose and raffinose were found to be up-regulated in foxtail millet under PEG stress.

#### DEGs involved in phenylpropanoids-related pathway and their down-regulated expression pattern in response to drought

**Fig. 3:**
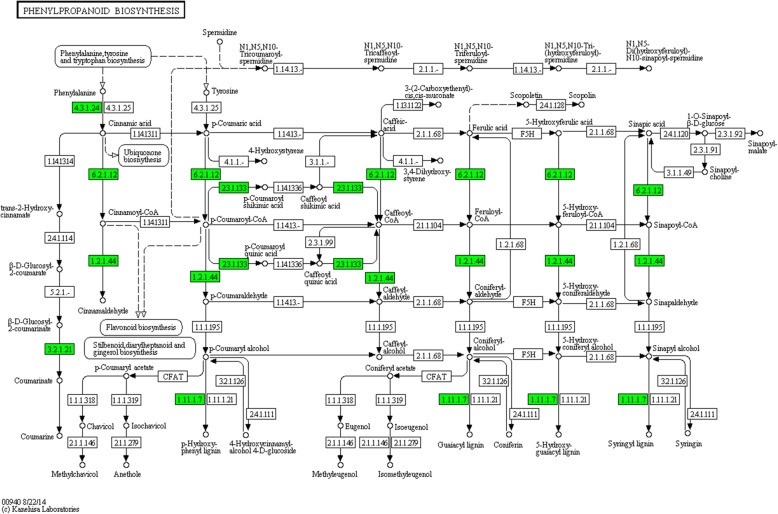
Coordinate down-regulation of genes involved in phenylpropanoid biosynthesis. The transcripts in phenylpropanoid biosynthesis pathway were down-regulated in CK14H vs. P14H. Green rectangle boxes indicated down-regulated genes

The metabolism of phenylalanine is an upstream pathway of phenylpropanoid. Phenylpropanoid biosynthesis provides the precursors for a wide range of phenolic compounds, such as ferulic acid, p-coumaric acid [15, 19]. During phase III, seventeen genes involved in phenylalanine metabolism were down-regulated in CK14H vs. P14H. The transcripts of 20 genes were depressed by PEG in the phenylpropanoid biosynthesis pathway (Fig. 3). After the duplication between two pathways was removed, 20 phenylpropanoids-related genes were found to be down-regulated in Jingu 20 under drought stress in phase III, which included 4-coumarate-CoA ligase 3, shikimate O-hydroxycinnamoyltransferase, cinnamoyl-CoA reductase 1, beta-glucosidase 6, phenylalanine ammonia-lyase and 15 peroxidases (Table 1). According to Fig. 3, the lower expression levels of these phenylpropanoids-related genes might affect the accumulation of phenolic compounds in the seeds under drought stress. And many phenylpropanoids and related metabolites have been reported to be allelochemicals and influence germination and growth of many plant, such as coumaric acid, ferulic acid [23]. Therefore, the expression patterns of phenylpropanoids-related DEGs, the contents and roles of different phenolic compounds deserve further analysis in foxtail milllet.

**Table 1:** Summaries of differentially expressed genes involved in phenylpropanoids-related pathway under PEG stress

Gene ID	CK2H vs. P2H (phase I)			CK8H vs. P8H (phase II)			CK14H vs. P14H (phase III)			Description
	Fold change (Log_2_ ratio)	UP/ DOWN	FDR	Fold change (Log_2_ ratio)	UP/ DOWN	FDR	Fold change (Log_2_ ratio)	UP/ DOWN	FDR	
Seita.1G065800	0.24	normal	1	−0.33	normal	0.05896	−1.04	down	6.63E-16	**4-Coumarate--CoA ligase 3 OS=*Oryza sativa subsp. japonica*(Rice)** (EC: 6.2.1.12)
Seita.1G240500	–	–	–	0.17	normal	1	−1.61	down	1.40E-07	**Phenylalanine ammonia -lyase OS=*Oryza sativa subsp. indica*(Rice)** (EC: 4.3.1.24)
Seita.1G361000	−0.06	normal	1	0.19	normal	1	−1.04	down	1.94E-06	**Cinnamoyl-CoA reductase 1 OS=*Arabidopsis thaliana*(Mouse-ear cress)** (EC: 1.2.1.44)
Seita.4G047200	−0.29	normal	1	−0.81	normal	0.15261	−1.07	down	7.49E-11	**Shikimate O-hydroxycinnamoyl-transferase OS=*Nicotiana tabacum*(Common tobacco)** (EC: 23.1.133)
Seita.9G492600	–	–	–	−0.51	normal	0.89386	−1.50	down	1.77E-08	**Beta-glucosidase 6 (Precursor) OS=*Oryza sativa subsp. japonica*(Rice)** (EC: 3.2.1.21)
Seita.1G023100	−0.15	normal	1	−0.66	normal	3.95E-08	−1.52	down	2.83E-33	**Peroxidase 52 (Precursor) OS=*Arabidopsis thaliana*** (EC: 1.11.1.7)
Seita.2G430900	–	–	–	−0.48	normal	0.90950	−1.68	down	5.45E-13	Peroxidase 70 (Precursor) OS=*Zea mays* (Maize) (EC: 1.11.1.7)
Seita.2G431100	–	–	–	−1.21	normal	0.22302	−1.70	down	1.36E-06	Peroxidase 66 (Precursor) OS=*Zea mays* (Maize) (EC: 1.11.1.7)
Seita.2G431200	–	–	–	−0.51	normal	1	−2.31	down	5.78E-11	Peroxidase 2 (Precursor) OS=*Oryza sativa subsp. japonica* (Rice) (EC: 1.11.1.7)
Seita.2G431300	–	–	–	–	–	–	−1.28	down	1.69E-06	Peroxidase 2 (Precursor) OS=*Oryza sativa subsp. japonica *(Rice) (EC: 1.11.1.7)
Seita.2G431500	–	–	–	–	–	–	− 1.59	down	0.00012	Peroxidase 66 (Precursor) OS=*Zea mays* (Maize) (EC: 1.11.1.7)
Seita.3G004800	−0.30	normal	1	−0.53	normal	0.22516	−2.05	down	5.87E-32	**Cationic peroxidase SPC4 (Precursor) OS=*Sorghum bicolor*(Sorghum)** (EC: 1.11.1.7)
Seita.3G234900	–	–	–	–	–	–	−4.21	down	0.00810	Peroxidase 15 (Precursor) OS=*Ipomoea batatas* (Sweet potato) (EC: 1.11.1.7)
Seita.3G235000	0.15	normal	1	−0.02	normal	1	−1.23	down	8.05E-11	Peroxidase 15 (Precursor) OS=*Ipomoea batatas* (Sweet potato) (EC: 1.11.1.7)
Seita.4G176700	−0.06	normal	1	−0.33	normal	0.86122	−1.61	down	6.80E-16	Peroxidase P7 OS=*Brassica rapa subsp. rapa* (Turnip) (EC: 1.11.1.7)
Seita.5G145500	–	–	–	−1.06	normal	0.01628	−1.08	down	4.17E-05	**Peroxidase 4 (Precursor) OS=*Vitis vinifera*(Grape)** (EC: 1.11.1.7)
Seita.7G271000	–	–	–	–	–	–	−2.07	down	1.49E-15	Peroxidase 1 (Precursor) OS=*Zea mays* (Maize) (EC: 1.11.1.7)
Seita.9G298300	–	–	–	–	–	–	−1.10	down	1.50E-06	Peroxidase 15 (Precursor) OS=*Ipomoea batatas* (Sweet potato) (EC: 1.11.1.7)
Seita.9G392700	–	–	–	–	–	–	−1.83	down	7.97E-11	Peroxidase 2 (Precursor) OS=*Zea mays* (Maize) (EC: 1.11.1.7)
Seita.9G478000	–	–	–	–	–	–	−1.37	down	2.72E-05	Peroxidase 15 (Precursor) OS=*Ipomoea batatas* (Sweet potato) (EC: 1.11.1.7)

Bold text indicated the phenylpropanoids-related genes which had been analyzed by qPCR in this research

### Expression analysis of DEGs related with phenylpropanoids pathway using qRT-PCR

**Fig. 4:**
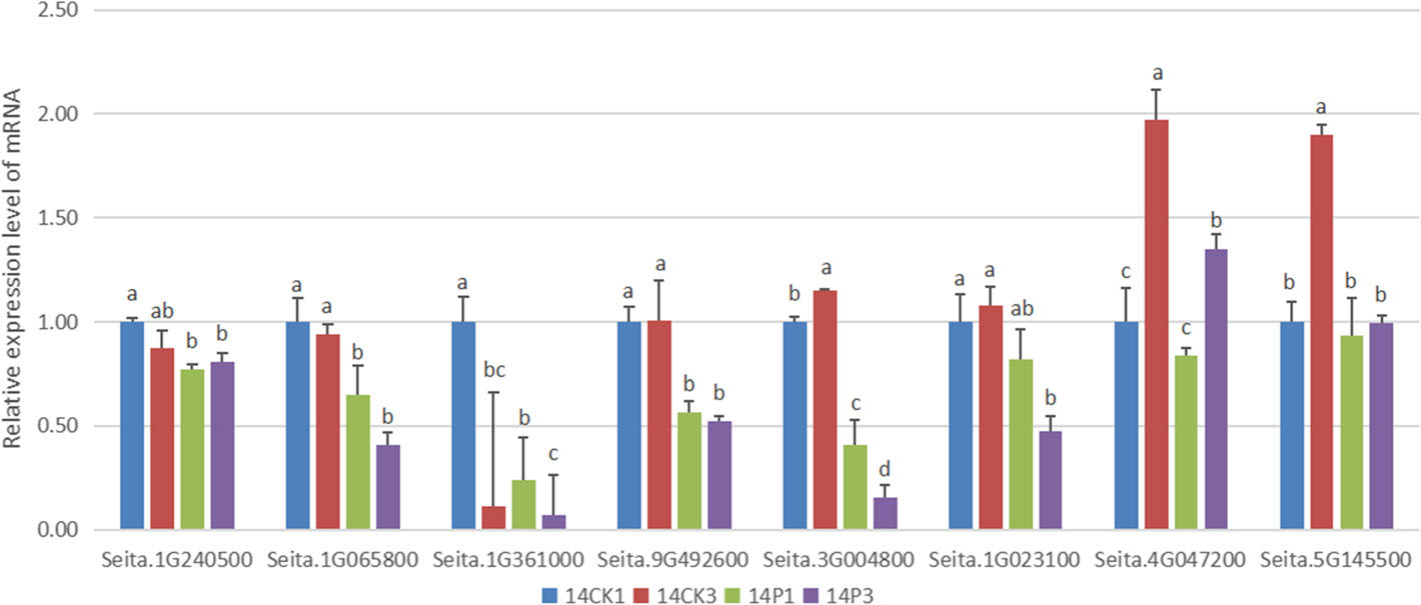
Validation of differential expression of drought responsive genes by qRT-PCR. The relative mRNA levels were normalized with the inner control gene (β-actin) and expressed relative to the corresponding value of 14CK1(control), which were given an arbitrary value of 1. **14CK1** indicated the foxtail millet transferred into another water pertri-dishes for 1 h after germinated for 14 h under normal conditions. **14CK3** represented the foxtail millet transferred into another water pertri-dishes for 3 h after germinated for 14 h under normal conditions. **14P1** indicated the foxtail millet treated by PEG stress for 1 h after germinated for 14 h under normal conditions. **14P3** represented the foxtail millet treated by PEG stress for 3 h after germinated for 14 h under normal conditions. The SD of different samples were calculated using one-way ANOVA followed by Tukey HSD multiple comparison

Since the most enriched pathway was “biosynthesis of phenylpropanoid”, eight DEGs encoding key enzymes in this pathway were selected for qRT-PCR validation (Table 1 and Fig. 4). In the sample of 14P1 (PEG treated for 1 h after germinating for 14 h under normal condition), the expression levels of Seita.1G240500, Seita.1G065800, Seita.1G361000, Seita.9G492600 and Seita.3G004800, decreased significantly compared with those in 14CK1 (germinating for 15 h under normal condition). While Seita.1G023100, Seita.4G047200 and Seita.5G145500 only showed similar expression trends. In 14P3 (PEG treated for 3 h after germinating for 14 h under normal condition), the genes, including Seita.1G065800, Seita.9G492600, Seita.3G004800, Seita.1G023100, Seita.4G047200 and Seita.5G145500, were dramatically down-regulated with 14CK3 (germinating for 17 h under normal condition) as control. While Seita.1G240500 and Seita.1G361000 just had decreased expression tendency. The comparison of qRT-PCR and RNA-seq assay data indicated a similar expression patterns in most of the selected DEGs except for the difference of fold change between them. The correlation between RNA-seq and qRT-PCR was analyzed in terms of fold changes. The Pearson coefficient was 0.54 (*p* value 0.03). These data supported the reliability of the sequencing results, and confirmed the down-regulated transcriptional expression of phenylpropanoids-related genes during seed germination under drought stress (Fig. 4).

**Fig. 5:**
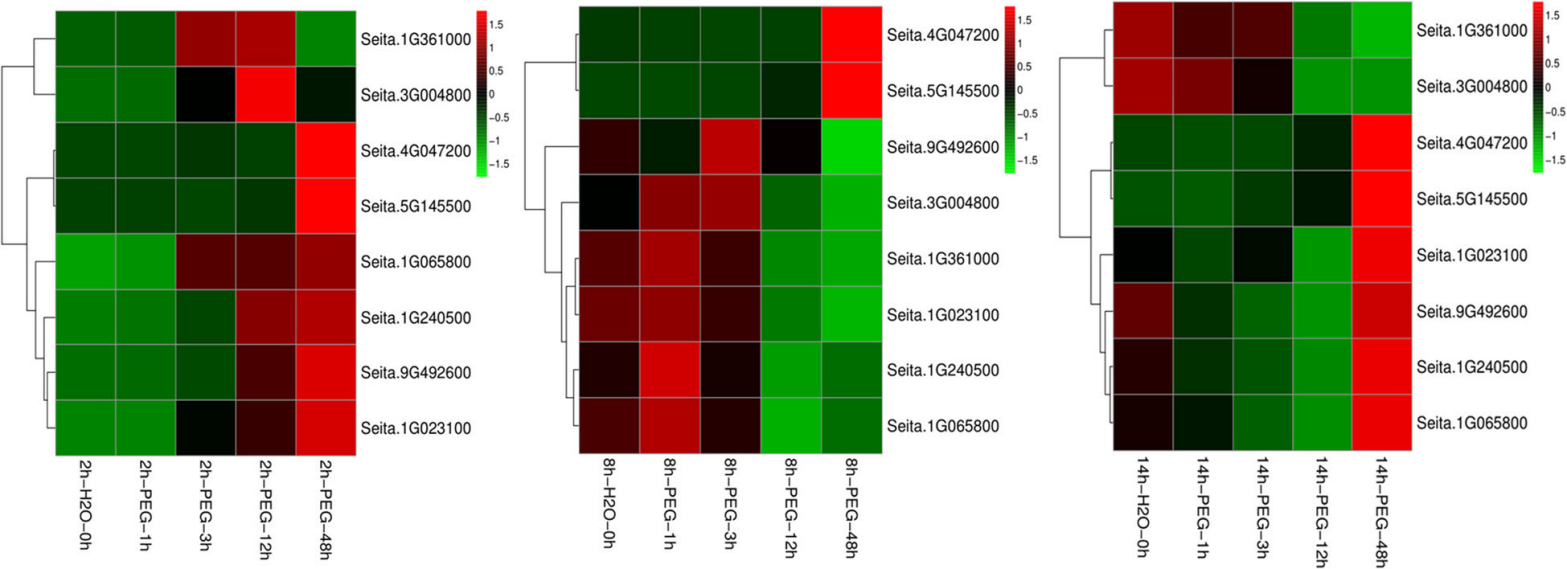
Expression analysis of drought responsive genes at different germination period under PEG stress by qRT-PCR. 2 h-H2O-0 h, 2 h-H2O-1 h, 2 h-H2O-3 h, 2 h-H2O-12 h and 2 h-H2O-48 h indicated respectively the foxtail millet seeds treated by PEG stress for 0 h, 1 h, 3 h, 12 h, 48 h, after germinated for 2 h under normal conditions. 8 h-H2O-0 h, 8 h-H2O-1 h, 8 h-H2O-3 h, 8 h-H2O-12 h and 8 h-H2O-48 h represented respectively the foxtail millet seeds treated by PEG stress for 0 h, 1 h, 3 h, 12 h, 48 h, after germinated for 8 h under normal conditions. 14 h-H2O-0 h, 14 h-H2O-1 h, 14 h-H2O-3 h, 14 h-H2O-12 h and 14 h-H2O-48 h indicated respectively the foxtail millet seeds treated by PEG stress for 0 h, 1 h, 3 h, 12 h, 48 h, after germinated for 14 h under normal conditions

To further verifying the down-regulated trends of DEGs in phenylpropanoids-related pathway during the germination period, eight key genes were analyzed by qRT-PCR at phase I (germinating for 2 h at normal condition), phase II (germinating for 8 h) and phase III (germinating for 14 h) under drought treatment (− 0.50 MPa PEG for 0 h, 1 h, 3 h, 12 h and 48 h) (Fig. 5). The results of qRT-PCR showed that the expression of phenylalanine ammonia-lyase (Seita.1G240500), 4-coumarate-CoA ligase 3 (Seita.1G065800), cinnamoyl-CoA reductase 1 (Seita.1G361000), beta-glucosidase 6 (Seita.9G492600), cationic peroxidase SPC4 (Seita.3G004800) and peroxidase 52 (Seita.1G023100) were up-regulated during phase I, while the expression of genes encoding shikimate O-hydroxycinnamoyl-transferase (Seita.4G047200) and peroxidase 4 (Seita.5G145500) only increased significantly at 48 h under PEG stress at phase I.

In phase II, the expression levels of four DEGs encoding 4-coumarate--CoA ligase 3 (Seita.1G065800), cinnamoyl-CoA reductase 1 (Seita.1G361000), cationic peroxidase SPC4 (Seita.3G004800) and peroxidase 52 (Seita.1G023100) decreased, while those of beta-glucosidase 6 (Seita.9G492600) were down-regulated only under PEG stress at 48 h. The expression of peroxidase 4 (Seita.5G145500) increased, whereas the expression levels of shikimate O-hydroxy- cinnamoyl-transferase (Seita.4G047200) were up-regulated only under PEG stress at 48 h. Additionally, the expression of gene encoding phenylalanine ammonia-lyase (Seita.1G240500) did not change obviously.

During phase III, the expression of five DEGs encoding phenylalanine ammonia-lyase (Seita.1G240500), 4-coumarate-CoA ligase 3 (Seita.1G065800), cinnamoyl-CoA reductase 1 (Seita.1G361000), beta-glucosidase 6 (Seita.9G492600) and cationic peroxidase SPC4 (Seita.3G004800) decreased under PEG stress, while the expression levels of shikimate O-hydroxycinnamoyl-transferase (Seita.4G047200) and peroxidase 4 (Seita.5G145500) were up-regulated, and the expression of peroxidase 52 (Seita.1G023100) remained unchanged. These results suggested that, the expression trends of phenylpropanoids related genes were different under drought stress during different germination period. And these genes probably play important roles in the regulation of germinating foxtail millet in response to drought stress.

### Metabolite accumulation pattern in phenylpropanoid pathway of foxtail millet

**Fig. 6:**
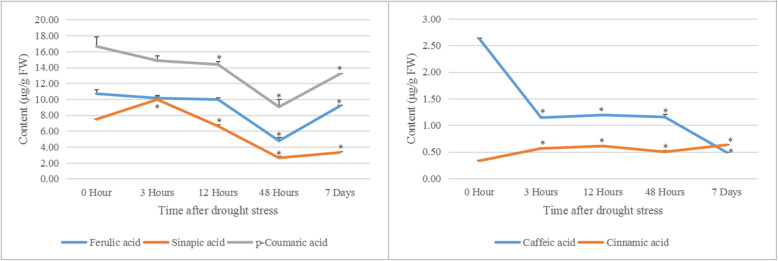
Content analysis of five phenylpropanoids-related metabolites in germinating seeds of foxtail millet treated by PEG. The contents of five metabolites (ferulic acid, sinapic acid, p-coumaric acid, caffeic acid and cinnamic acid) were analyzed in foxtail millet seeds, which grew for 14 h under normal conditions and then treated by PEG for 3 h, 12 h, 48 h and 7 d. The mean values and SD of metabolite contents were calculated using one-way ANOVA followed by Tukey HSD multiple comparison(*P < 0.05)

Five phenylpropanoids-related metabolites were analyzed in germination seeds of millet treated by PEG at 14 h after growing under water condition. The results shown that the higher amount of cinnamic acid was accumulated in germinating seeds under PEG than that in the control. The levels of sinapic acid increased at 3 h, but decreased at 12 h, 48 h and 7 days under PEG conditions. However, the contents of p-coumaric acid and ferulic acid decreased during stress. The contents of caffeic acid declined significantly under PEG stress (Fig. 6).

### The effects of phenolic compounds on foxtail millet germination

**Fig. 7:**
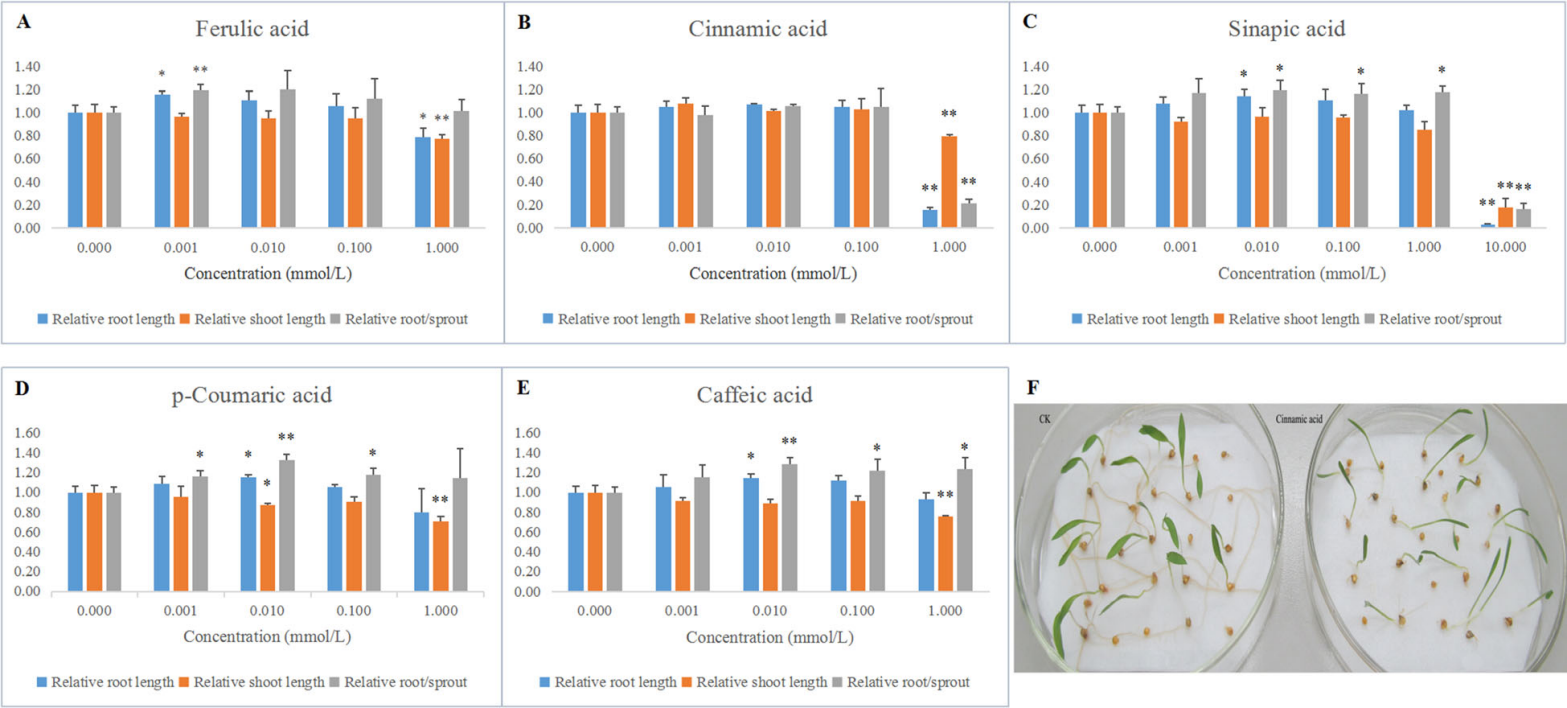
The effects of five phenolic compounds on the germination of foxtail millet. **a**, **b**, **c**, **d**, **e** indicated the effects of five phenolic compounds on the germination of foxtail millet respectively. **f** represented the phenotypic symptoms of cinnamic acid affecting the germination of foxtail millet . The mean values and SD in (**a**), (**b**), (**c**), (**d**) and (**e**) were calculated using Student’s *t*-test (**P < 0.01, *P < 0.05)

The five phenolic compounds were bioassayed for their effects on germination and growth of foxtail millet (Fig. 7). The analysis results indicated that the germination of foxtail millet was completely inhibited at 10 mM concentration of ferulic acid, p-coumaric acid, cinnamic acid and caffeic acid. The 10 mM concentration of sinapic acid dramatically reduced shoot and root growth. Ferulic acid, p-coumaric acid, cinnamic acid and caffeic acid remarkably inhibited shoots growth at 1 mM concentration. In particular, cinnamic acid more obviously restrained the growth of roots than the growth of shoots at 1 mM concentration (Fig. 7b). By contrast, cinnamic acid slightly promoted shoot growth at at 10^− 6^ M, 10^− 5^ M and 10^− 4^ M concentration. The 10^− 6^ M concentration of ferulic acid apparently promoted root growth and the root/sprout in this experiment. Sinapic acid, caffeic acid and p-coumaric acid separately promoted root growth at 0.01 mM concentration. Sinapic acid and caffeic acid helped noticeably the growth of root/sprout at concentrations of 0.01 M, 0.1 M and 1 mM. p-Coumaric acid promoted the root/sprout at 10^− 6^ M, 10^− 5^ M and 10^− 4^ M concentration.

## Discussion

Foxtail millet is known as a relatively drought-tolerant crop, which needs less water in germination period than the other cereal crops [14]. Germination and growth of seeds depend on the germination conditions. Under 24 °C ~ 26 °C conditions in our culture room, the water uptake pattern of foxtail millet seeds was explored during germination period, which included phase I (from 0 to 6 h after sowing), phase II (from 6 to 12 h after sowing) and phase III (commencing at 12 h after sowing)(Fig. 1), which was consistent with the previous report [5].

According to three phases, twelve cDNA libraries have been sequenced in germinating seeds to gain a global view of the drought induced changes and germination regulation pattern at transcriptome level, and responsive metabolic pathways. Moreover, the stress time was very short, such as 1 h and 3 h. In response to PEG stress, total 302 up-regulated and 355 down-regulated genes were identified between CK14H and P14H in phase III, 24 up-regulated and 18 down-regulated genes between CK8H and P8H in phase II, and 3 up-regulated and 3 down-regulated genes between CK2H and P2H in phase I (Additional file 3: Figure S3). These results indicated that foxtail millet was more sensitive to drought during the further increase in water uptake period than in the rapid initial uptake and plateau period. Thus the subsequent analysis emphases were laid on the further increase in water uptake period.

Using KEGG enrichment, the pathways of phenylpropanoid biosynthesis, plant hormone signal transduction and phenylalanine metabolism were highly enriched in CK14H vs. P14H (Fig. 2). These findings suggested that many phenylpropanoids-related genes played important roles in drought response during phase III of foxtail millet. Similarly, the DEGs of germinating millet under drought stress for 10 h and 18 h were found to be associated with phenylpropanoids metabolism and plant hormone signal transduction [32]. The changes of transcript levels of phenylpropanoids-related genes were found in *Ricinus communis* under low temperature [8]. Soybean genes in the phenylpropanoid synthesis pathway were up-regulated in both alkaline and low Fe conditions [33].

Phenylalanine metabolism provides the precursors for the synthesis of phenylpropanoid [15]. In our study, 17 genes involved in phenylalanine metabolism were down-regulated under PEG stress, and the transcripts of 20 genes were decreased in the phenylpropanoid biosynthesis pathway in CK14H vs. P14H (Fig. 3). A total of 20 phenylpropanoids-related genes were found to be down-regulated in foxtail millet under stress (Table 1). The identified phenylpropanoids-related genes were phenylalanine ammonia-lyase, 4-coumarate--CoA ligase 3, shikimate O-hydroxycinnamoyltransferase, cinnamoyl-CoA reductase 1, beta-glucosidase 6 and fifteen peroxidase. Phenylalanine ammonia lyase (PAL, EC4.3.1.24), the first enzyme in the phenylpropanoid biosynthesis pathway, catalyzes the conversion of L-phenylalanine to ammonia and trans-cinnamic acid [19]. PAL is an inducible enzyme that responds to biotic and abiotic stresses, including pathogen infection, wounding, nutrient depletion, UV irradiation, and extreme temperatures [17, 20, 22, 34–39]. PAL plays an important role in plant defense, which is involved in the biosynthesis of salicylic acid (SA) [40–42]. 4-Coumarate--CoA ligase and cinnamoyl-CoA reductase (CCR) are important enzymes in catalyzing the conversion of coumaric acid, caffeic acid, ferulic acid, 5-hydroxyferulic acid and sinapic acid to lignin [43–52]. And many phenylpropanoids and their metabolites are known allelochemicals, for example cinnamic acid, p-coumaric acid, caffeic acid, ferulic acid and sinapic acid, which have been reported to prohibit the germination of many plant seeds [23]. Therefore, these results indicated that the lower expression levels of genes in phenylpropanoid biosynthesis pathway might lead to the accumulation changes of allelochemicals in foxtail millet seeds, thus regulating the seed germination and seedling growth under drought stress.

To verify the expression pattern of these phenylpropanoids-related genes, eight key gene in phenylpropanoids-related pathway were analyzed by qRT-PCR at phase I (germinating for 2 h), phase II (germinating for 8 h), phase III (germinating for 14 h) under PEG treatment. These results showed that during different germination period, the expression trends of phenylpropanoids related genes were different under drought stress, respectively. For instance, when the seeds suffered from drought during phase I, the expression levels of these genes encoding phenylalanine ammonia-lyase (Seita.1G240500), 4-coumarate--CoA ligase 3 (Seita.1G065800), cinnamoyl-CoA reductase 1 (Seita.1G361000), beta-glucosidase 6 (Seita.9G492600) and cationic peroxidase SPC4 (Seita.3G004800) would be up-regulated, while the expression levels of these genes were down-regulated during phase III. In phase II, only 4-coumarate--CoA ligase 3 (Seita.1G065800), Cinnamoyl-CoA reductase 1 (Seita.1G361000) and Cationic peroxidase SPC4 (Seita.3G004800) were decreased. These results implied that the phenylpropanoids-related pathway played different roles in the regulation of foxtail millet in response to drought stress during different germination periods. In other words, the expression changes of phenylpropanoids related genes might increase or reduce the concentrations of phenylpropanoids related metabolites in the germination seeds, and thus result in promoting and inhibiting the germination and growth of foxtail millet under drought stress in different germination stages.

Water stress caused by osmotic stress or drought, can stimulate accumulation of allelochemicals [53]. To confirm the accumulation changes of these phenylpropanoids-related metabolites, five metabolites related to phenylpropanoids were analyzed in germination seeds of foxtail millet. The analysis shown that the higher amount of cinnamic acid was accumulated in germinating seeds under PEG stress than that in the control. However, the contents of p-coumaric acid, caffeic acid, ferulic acid and sinapic acid were decreased. This is consistent with the conclusion that drought caused the increase of ferulic acid content in wheat [54].

The effects of the five phenolic compounds on germination and growth of foxtail millet were tested by external application. The highest concentrations of five phenolic compounds remarkably inhibited the germination or the growth of shoots and roots. In particular, cinnamic acid apparently restrained the growth of roots more than the growth of shoots at 1 mM concentration. The lower concentrations of ferulic acid, sinapic acid, caffeic acid and p-coumaric acid promoted root growth and root/sprout. They would jointly play roles at the same times and keep a coordination among themselves. Combined with the concentration changes of five metabolites in phenylpropanoids-related pathway under PEG stress, the content of cinnamic acid was dramatically increased and the amounts of ferulic acid, sinapic acid, caffeic acid and p-coumaric acid decreased in germinating seeds. These results implied that phenolic compounds would regulate the growth of foxtail millet under drought conditions. Accordingly, from phenotypic symptoms, drought stress slightly affected the growth of roots, while significantly reduced the length of shoots. Similar observations have been reported in *Arabidopsis thaliana* [23]. Ferulic acid, p-coumaric acid and sinapic acid showed inhibitory effect on the germination and radicle length of *A. thaliana*. Ferulic acids inhibited total germination of *A. thaliana* above 500 and 750 μM. Wu et al. found that ferulic acids had no effect on annual bluegrass germination [55]. Reigosa et al. reported that lower concentrations of phenolic compounds stimulated the growth of six weeds or had no actions [56]. Ferulic and p-coumaric acids had inhibitory effects on root growth of *A. thaliana* without nutrients [23]*.* And it was found that in tobacco p-coumaric inhibited root growth while ferulic acid had stimulatory effects [57]. These results all indicate that various plant show different response to allelochemicals [58].

Due to lack of motility and immune system, plants have developed their defense strategies in the process of evolution, for example, the production of secondary metabolites as a tool in order to adapt to the changing environment and overcome stress constraints [59]. These allelochemicals act as first line of defense against abiotic stresses [60, 61]. In particular, many desert plants have adapted to the harsh environment through secondary metabolites. Allelopathy of these chemicals can also affect neighboring plants [62]. Allelopathy activity from plants grown in dry soils was greater than that provoked from plants grown in well-watered soils. Under water stress conditions, the donor plants contained a greater amount of allelopathic chemicals per dry weight than in absence of water stress, and the growth of target plants was reduced. This fact could cause autotoxicity problems in natural conditions [53, 63]. From the above results, foxtail millet has probably developed defense strategies in response to drought stress based on phenylpropanoids-related metabolites as desert plant, and avoided drought by their allelopathy and autotoxicity roles. Of course, these need further detailed experimental verification in the future.

In addition, in this study, many DEGs related to transport, signal transduction, phytohormone and osmotic homeostasis were identified in Jingu 20 during different germination periods under PEG stress. In CK14H vs. P14H, 37 transporters, 38 protein kinases, 2 PYL, 12 phosphatases, 2 JAZ, 56 transcription factors, 13 genes involved in phytohormone biosynthesis and 18 genes related to osmo-protention were differentially expressed under drought stress. These results showed that foxtail millet was in response to drought stress and modulating stress tolerance during germination period. With regard to the relation between phenylpropanoids-related pathway and the above pathways, it was only known that phenylalanine metabolism was upstream of salicylic acid and phenylpropanoid biosynthesis (Additional file: Figure S7). The knowledge of its mechanisms was largely unknown and needed to be further studied in foxtail millet.

## Conclusions

The transcriptomic results demonstrate differentially expressed genes related to phenylpropanoids pathway involved in foxtail millet drought resisting. There were remarkable decreases in the expressions of phenylpropanoids-related genes during the further increase in water uptake period (phase III) of germination under PEG stress, accompanied by an increase of cinnamic acid, and reductions in p-coumaric acid, caffeic acid, ferulic acid and sinapic acid. The higher concentrations of external five phenolic acid inhibited seeds germination or the growth of shoots and roots, but the lower concentrations promoted the growth of seedlings or had no effects. The 1 mM concentration of cinnamic acid more obviously restrained the growth of roots than the growth of shoots. Ferulic acid, caffeic acid, sinapic acid and p-coumaric acid could increase the root length and root/sprout in lower concentration. Thus these findings indicate that foxtail millet could avoid drought by regulation of allelochemical concentrations. Allelochemicals would be accumulated and reduced by regulating the gene expressions of phenylpropanoids-related pathway during germination. These integrative results would provide valuable information for understanding the process of drought resistance during in foxtail millet germination, which would be benefit to foxtail millet cultivar breeding and innovation, promoting germination under drought stress, and seed priming.

## Materials and methods

### Plant materials and cultivar screening by PEG treatment

All foxtail millet cultivars were obtained from and preserved in Millet Research Institute of Shanxi Academy of Agricultural Sciences, which were planted every three years. We undertook the formal identification of the plant materials by phenotype and stress resistance. The voucher specimens of foxtail millet cultivars in this research were deposited in Physiological and Biochemical Laboratory. The deposition numbers of Yugu1, An04–4783, Changnong35, Jingu20, Jingu34, Jingu9, Tieganhan, Lugu6 were si-2015-b13, si-2015-358, si-2015-g54, si-2015-g1, si-2015-g3, si-2015-b15, si-2015-b19, si-2015-b21, respectively. We evaluated the cultivar variations of the 8 foxtail millet genotypes in the context of early drought tolerance using polyethylene glycol (PEG) induced osmotic stress at the germination and early seedling growth stage. The stress was imposed by exposing the germinating grain to polyethylene glycol. The osmotic potential of polyethylene glycol 6000 was − 0.50 MPa [64]. The germination percentage, the relative sprout length and the relative root length were observed against the controls. From these screening, the cultivar of highest drought resistance was selected to understand the phenotypic and molecular basis of tolerance mechanisms.

### Water uptake pattern of foxtail mellit seeds during germination stage

Fifty seeds of the selected cultivar were allowed for germination under water in pertri-dishes, each was replicated three times. Seeds germinated during June–August when the temperature remained between 24 °C ~ 26 °C in culture room. Their fresh weights were measured every one hour by drying them with absorbent paper during germination period in foxtail millet. Then the growth rate of seed fresh weight was calculated against dry seed weight. The map was draw by the growth rate of seed fresh weight.

### Effect of PEG stress during germination period and sample preparation

Seeds of the selected cultivar were surface-sterilized in 3% sodium hypochloride for 20 min and rinsed 5 times (4 min/each) with distilled water. These seeds were allowed for germination in 9 cm petri-dishes at room temperature ranged from 24 °C to 26 °C and relative humidty ranged from 40 to 50% in the culture room. Each was replicated three times. After germinating for 2 h, 8 h and 14 h (including the time of sterilizing and growing) under normal conditions, the seeds were transplanted in plates containing polyethylene glycol 6000 solution. The osmotic potential of polyethylene glycol 6000 was − 0.50 MPa. The control plants were cultured and transferred in the same ways as the dehydration treatments, but without the addition of PEG. The shoot length and root length of foxtail millet stressed by PEG were measured on the 7th day of germination. Under the same conditions, the germinating seeds used for sequencing were collected, which were CK2H, CK8H, CK14H (germinating respectively for 2 h, 8 h, 14 h under normal condition respectively, then growing for 1 h and 3 h without PEG stress treatment), and P2H, P8H, P14H (germinating separately for 2 h, 8 h, 14 h under normal condition respectively, then growing for 1 h and 3 h under PEG treatment). Three biological replicates were set at the same time. Each biological duplication was carried out every 48 h. Three biological repeats were mixed into one sample. The experiments were repeated twice for collecting the sequencing samples. The first repeated samples were − 1 of sequencing samples, and the second repeated ones were − 2 of sequencing samples. The seeds treated by PEG were sampled at the times (1 h, 3 h, 12 h and 48 h) respectively. At the same time, the seeds growing at normal condition were collected during germination phases for expression analysis by qRT-PCR. The collected samples were frozen in liquid nitrogen and stored at − 80 °C for RNA Extraction.

### RNA extraction, library preparation, and transcriptome sequencing

Frozen seed samples were ground in liquid nitrogen and total RNA was extracted using CTAB methods. RNA degradation and contamination were monitored on 1% agarose gels. RNA purity was checked using the NanoPhotometer® spectrophotometer (IMPLEN, CA, USA). RNA integrity was assessed using the RNA Nano 6000 Assay Kit of the Agilent Bioanalyzer 2100 system (Agilent Technologies, CA, USA). The transcriptome cDNA libraries were prepared using NEBNext®Ultra™ RNA Library Prep Kit (NEB, USA) and purified with AMPure XP system (Beckman Coulter, Beverly, USA) according to manufacturer’s protocols. Sequencing was performed on an Illumina Hiseq 2500 following manufacturer’s recommendations (Beijing, China).

### Data analysis

After RNA sequencing, the raw data of fasta format were firstly processed through in-house perl scripts. Clean reads were obtained by removing reads containing adapter, reads containing ploy-N and low quality reads from raw data. Q30 and GC-content of the clean data were calculated. The clean reads were mapped and annotated based on the reference genome. Tophat2 was used as the tool soft for maping [65]. Read count for each gene was obtained from the mapping results. Gene expression levels were estimated by RSEM for each sample [66]. DESeq R package was used to analyze differential expression of two groups [67]. *P* value corrections were performed using the Benjamini and Hochberg’s approach. The genes with corrected *P*-value less than 0.05 were considered differentially expressed.

### Gene functional annotation

Gene function was annotated based on the following databases: Nr (NCBI non-redundant protein sequences), Nt (NCBI non-redundant nucleotide sequences), Pfam (Protein family), COG (Clusters of orthologous groups of proteins), Swiss-Prot (A manually annotated and reviewed protein sequence database), KO (KEGG ortholog database) and GO (Gene ontology). The enrichment of differential expression genes in KEGG pathways was analyzed using KOBAS software [68].

### Validation of differential expression using qRT-PCR

Expression of eight genes involved in phenylpropanoids-related pathway under PEG stress were examined by qRT-PCR analysis using SYBR *Premix Ex Taq* II (TaKaRa, Dalian, China) on Thermal Cycler Dice Real Time System (TaKaRa Code.TP800, Japanese). The PCR reactions were performed according to the manufacturer’s protocol. Each 25 μL amplification reaction contained 12.5 μL of SYBR *Premix Ex Taq* II (TaKaRa, Dalian, China), 1 μL of each primer (10 μM), 8.5 μL of sterile distilled water, and 2 μL of cDNA template. The PCR reaction contained an initial denaturation (95 °C for 30 s) followed by 40 cycles of 95 °C denaturation for 5 s and 60 °C annealing for 30 s. Gene specific primers were designed according to non-conserved region sequences of each gene using Primer Premier 5.0 software. Gene-specific primers were listed in Table S2. *β-Actin* gene of foxtail millet (Seita.7G294000) was used as internal controls for normalization as described by Xu [69]. The relative expression levels of the genes under PEG stress during different stages were calculated via the double standard curve method [70].

### Determination of metabolite content in phenylpropanoid biosynthesis of foxtail millet

The contents of cinnamic acid, p-coumaric acid, caffeic acid, ferulic acid and sinapic acid in germinating seeds were analyzed by HPLC [71]. The seeds of foxtail millet germinated for 14 h under water conditions, then grew for 3 h, 12 h, 48 h and 7 days under PEG treatment (− 0.50 MPa).

### The effects of phenolic compounds on the germination of foxtail millet

Cinnamic acid, p-coumaric acid, caffeic acid, ferulic acid and sinapic acid were tested for their effects on the germination and growth of foxtail millet. Five phenolic compounds were prepared in concentrations of 10 mM, 1 mM, 0.1 mM, 0.01 mM and 0.001 mM [56]. Solutions were bioassayed on seeds of foxtail millet. Twenty five seeds were placed on Whatman 3 MM paper in 9 cm diameter Petri dishes, to which 7 ml of solutions were add at the beginning and two additional ml of each solution were added on the third day. Three replicates of each treatment were incubated in the germination chamber under the same conditions as mentioned above.

## Supplementary information

**Additional file 1: Figure S1**. Differential responses of foxtail millet cultivars to PEG stress during germination stage. Phenotypes of eight foxtail millet cultivars were observed on the seventh day under PEG conditions during germination period. The cultivars in first line from left to right were Yugu1, An04–4783, Changnong35 and Jingu20, respectively. The genotypes in second line from left to right were Jingu34, Jingu9, Tieganhan and Lugu6, respectively.

**Additional file 2: Figure S2**. Germination rates of foxtail millet cultivars under PEG stress during germination period. Germination rates of Yugu1, An04–4783, Changnong35, Jingu20, Jingu34, Jingu9, Tieganhan and Lugu6 were counted on the 7th day under PEG conditions. The mean values and SD were calculated using one-way ANOVA followed by Tukey HSD multiple comparison.

**Additional file 3: Figure S3**. Volcano plot of genes with differential expression between control and treated sample libraries. The X-axis indicated the log2(FC) of DEGs (FC, fold change). The Y-axis represented the -log10(FDR) of differential expression genes (FDR, False Discovery Rate). Red dots indicated up-regulated genes. Green dots represented down-regulated genes, and black dots indicated non-different expression genes. DEGs between control and PEG stress were identified in different germination periods according to a threshold of fold change ≥2 and FDR ≤0.01 (FDR, false discovery rate). A, DEGs of CK2H vs. P2H. (n = 6; 3 up and 3 down) B, DEGs of CK8H vs. P8H. (n = 42; 24 up and 18 down) C, DEGs of CK14H vs. P14H (n = 657; 302 up and 355 down).

**Additional file 4: Figure S4**. COG classifications of DEGs of foxtail millet in CK14H vs. P14H. A total of 228 DEGs in CK14H vs. P14H were assigned to 21 COG categories. The capital letters on the x-axis indicated the COG categories as listed on the right of the histogram. The y-axis represented the percentages of the corresponding category among all categories. The number of DEGs belong to the category, and the percentages of the corresponding category among all categories were separately indicated in square brackets.

**Additional file 5: Figure S5**. KEGG classifications of DEGs of foxtail millet in CK14H vs. P14H. The x-axis indicated the percentages of DEGs among the total annotated genes. The left y-axis represented the pathways. The number of DEGs belonged to the annotated pathway were represented after the histograms.

**Additional file 6: Figure S6**. The pathways with the most significant Q value in CK8H vs. P8H. The x-axis indicated rich factor of DEGs belong to the corresponding pathway. The left y-axis represented the pathways. The sizes of bubble represented the number of DEGs in the corresponding pathway, and the colors of the bubble represented the enrichment Q value of the corresponding pathway.

**Additional file 7: Figure S7**. Schematic representation of PEG stress signal transduction pathway during germination period of foxtail millet.

**Additional file 8: Table S1** - Summary of the sequencing and the reads mapping from the control groups (CK) and the PEG stress groups (P). The sequencing samples were CK2H (germinating for 2 h under normal conditions, then growing for 1 h and 3 h without PEG stress treatment), P2H(germinating for 2 h, then growing for 1 h and 3 h under PEG treatment), CK8H (germinating for 8 h under normal conditions, then growing for 1 h and 3 h without PEG stress treatment), P8H(germinating for 8 h under normal conditions, then growing for 1 h and 3 h under PEG treatment), CK14H(germinating for 14 h under normal conditions, then growing for 1 h and 3 h without PEG stress treatment), P14H (germinating for 14 h under normal conditions, then growing for 1 h and 3 h under PEG treatment). -1 represented the first repeat. -2 represented the second repeat.

**Additional file 9: Table S2**. Primer sequences of genes used for qRT-PCR.

**Additional file 10: Table S3**. DEGs during different germination periods of foxtail millet under PEG stress. DEGs of CK14H vs. P14H, CK8H vs. P8H and CK2H vs. P2H were identified at a threshold of fold change ≥2 and FDR ≤0.01 (FDR, false discovery rate).

**Additional file 11: Table S4**. Significant DEGs involved in PEG stress during different germination periods. DEGs related with transporters, signal transduction components, transcription factors, phytohormones biosynthesis and osmotic homeostasis were selected according to gene functions and metabolic pathways.

## Data Availability

The RNA-Seq data has been deposited in the Sequence Read Archive (SRA) at the National Center for Biotechnology Information (NCBI). The accession number is PRJNA573803, which includes 12 accession items (SRX6967585, SRX6967584, SRX6967583, SRX6967582, SRX6967581, SRX6967580, SRX6967579, SRX6967578, SRX6967577, SRX6967576, SRX6967575, SRX69675794).

## Abbreviations

PEG: Polyethylene glycol
DEGs: Differentially expressed genes,
FDR: False discovery rate
COG: Clusters of orthologous groups
KEGG: Kyoto encyclopedia of genes and genomes
qRT-PCR: Quantitative real time polymerase chain reaction
M: mol/L
cDNA: Complementary DNA
PAL: Phenylalanine ammonia lyase
CCR: Cinnamoyl-CoA reductase
RNA: Ribonucleic acid
CTAB: Cetyltrimethylammonium ammonium bromide
FC: Fold-change
FPKM: Fragments per kilobase of exon model per million mapped reads
GO: Gene ontology
PCR: Polymerase chain reaction
RNA-Seq: RNA sequencing
HPLC: High performance liquid chromatography
