# Supplementary figures and images for: Transcriptome and metabolite analysis reveal the drought tolerance of foxtail millet significantly correlated with phenylpropanoids-related pathways during germination process under PEG stress
Source: BMC Plant Biol. 2020 Jun 15;20:274. doi: 10.1186/s12870-020-02483-4 (PMC7296958; doi:10.1186/s12870-020-02483-4)

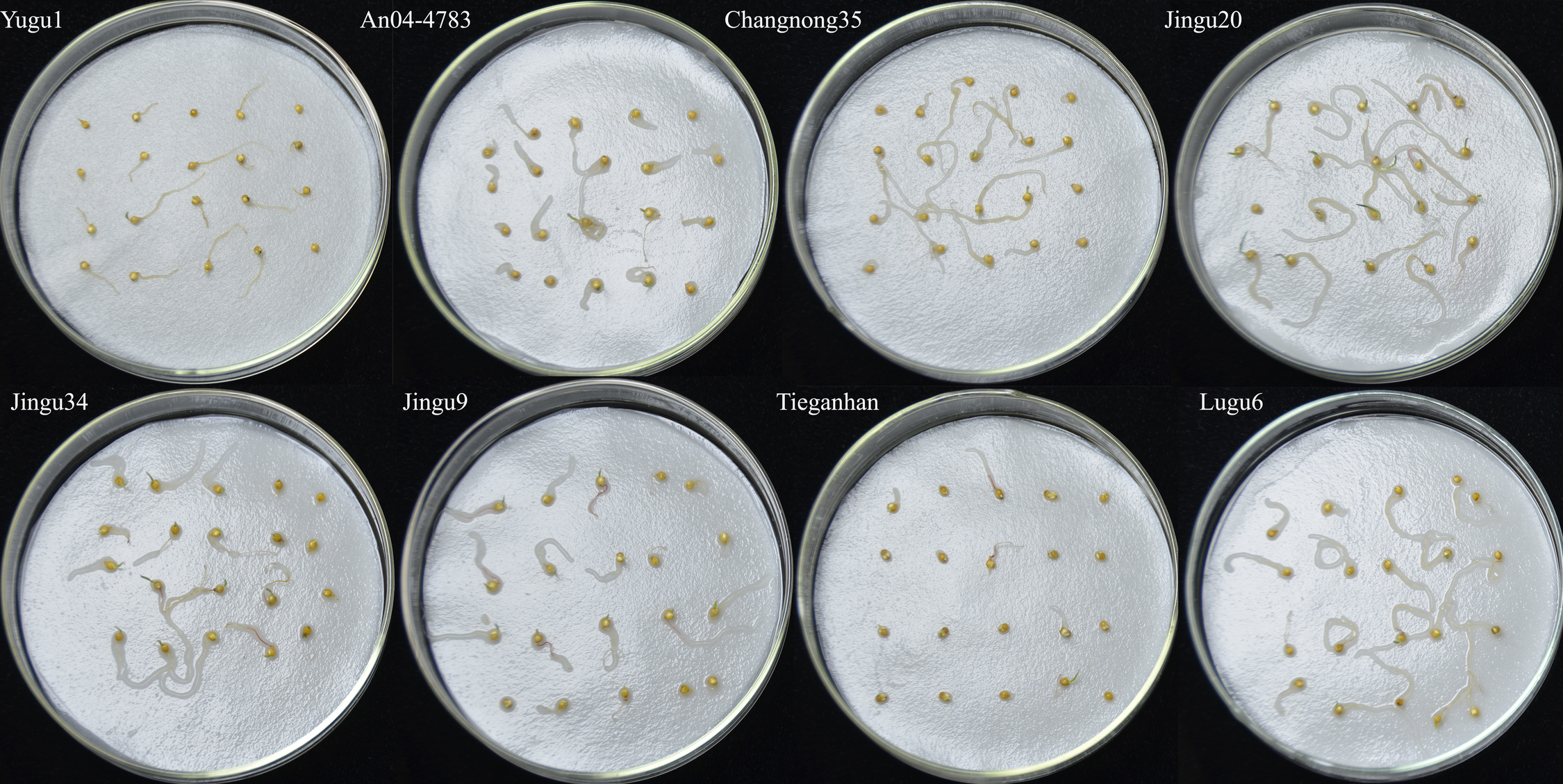

Supplement: Supplementary file 1 — Additional file 1: Figure S1. Differential responses of foxtail millet cultivars to PEG stress during germination stage. Phenotypes of eight foxtail millet cultivars were observed on the seventh day under PEG conditions during germination period. The cultivars in first line from left to right were Yugu1, An04–4783, Changnong35 and Jingu20, respectively. The genotypes in second line from left to right were Jingu34, Jingu9, Tieganhan and Lugu6, respectively. [file 12870_2020_2483_MOESM1_ESM.tif]

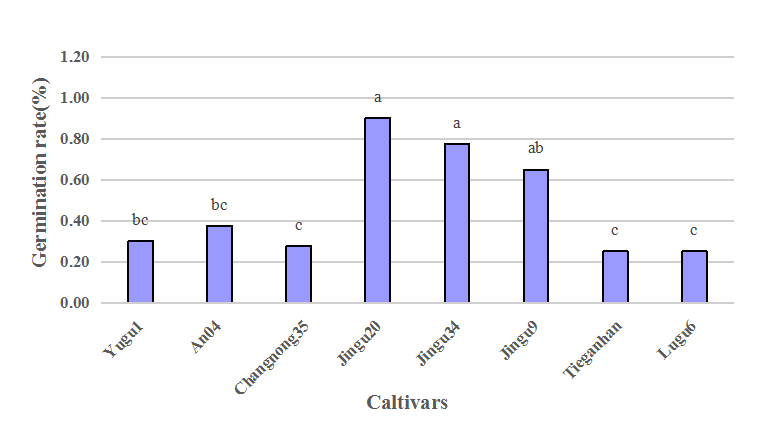

Supplement: Supplementary file 2 — Additional file 2: Figure S2. Germination rates of foxtail millet cultivars under PEG stress during germination period. Germination rates of Yugu1, An04–4783, Changnong35, Jingu20, Jingu34, Jingu9, Tieganhan and Lugu6 were counted on the 7th day under PEG conditions. The mean values and SD were calculated using one-way ANOVA followed by Tukey HSD multiple comparison. [file 12870_2020_2483_MOESM2_ESM.tif]

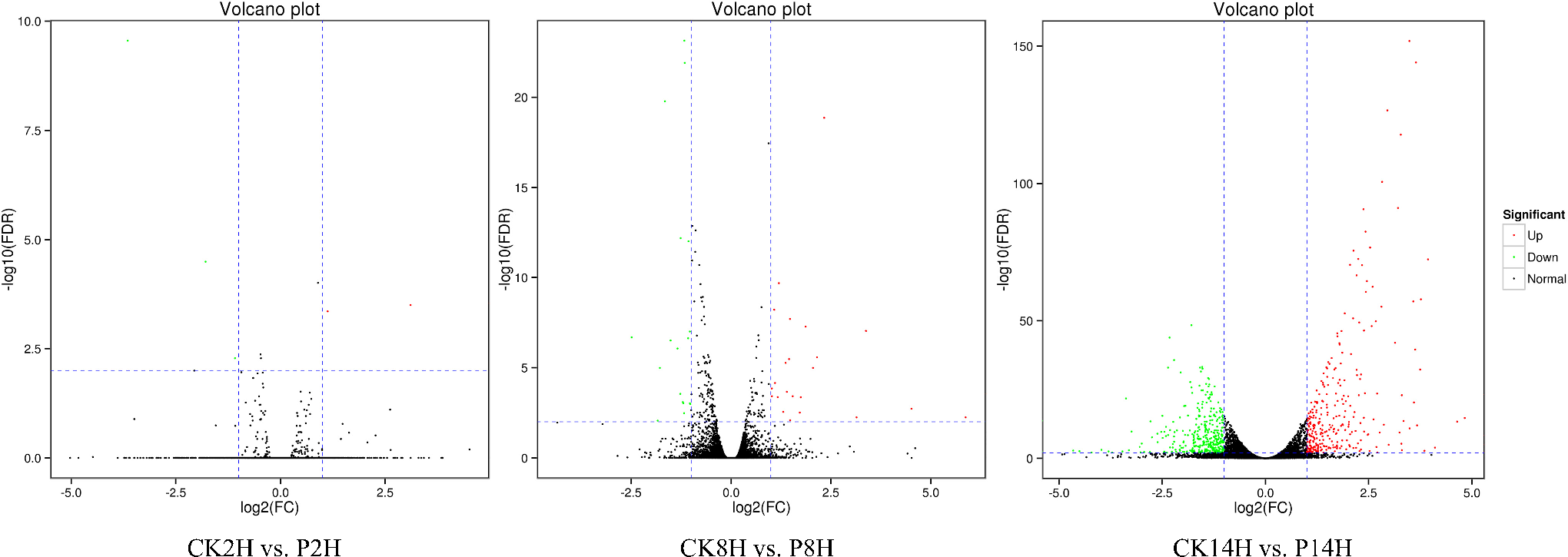

Supplement: Supplementary file 3 — Additional file 3: Figure S3. Volcano plot of genes with differential expression between control and treated sample libraries. The X-axis indicated the log2(FC) of DEGs (FC, fold change). The Y-axis represented the -log10(FDR) of differential expression genes (FDR, False Discovery Rate). Red dots indicated up-regulated genes. Green dots represented down-regulated genes, and black dots indicated non-different expression genes. DEGs between control and PEG stress were identified in different germination periods according to a threshold of fold change ≥2 and FDR ≤0.01 (FDR, false discovery rate). A, DEGs of CK2H vs. P2H. (n = 6; 3 up and 3 down) B, DEGs of CK8H vs. P8H. (n = 42; 24 up and 18 down) C, DEGs of CK14H vs. P14H (n = 657; 302 up and 355 down). [file 12870_2020_2483_MOESM3_ESM.tif]

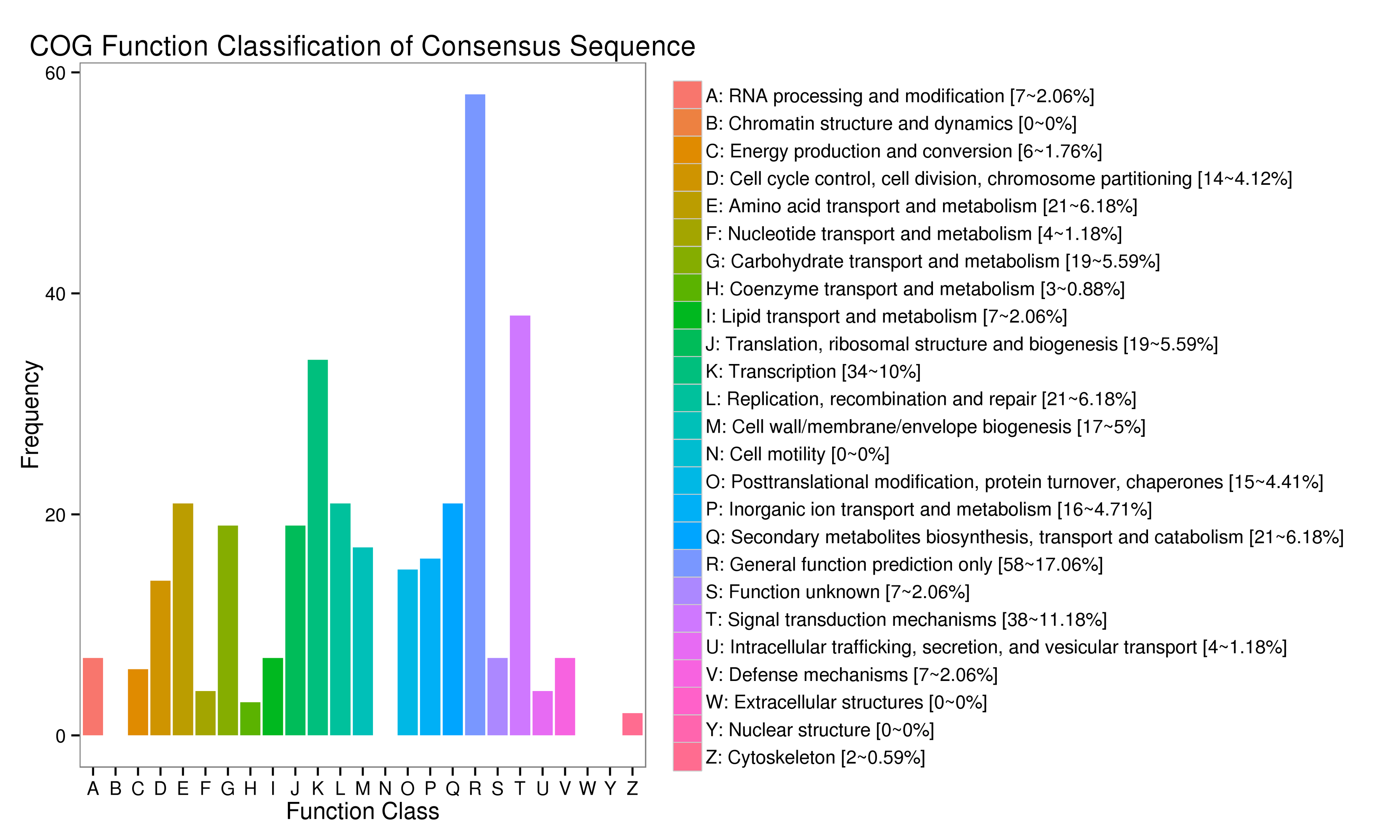

Supplement: Supplementary file 4 — Additional file 4: Figure S4. COG classifications of DEGs of foxtail millet in CK14H vs. P14H. A total of 228 DEGs in CK14H vs. P14H were assigned to 21 COG categories. The capital letters on the x-axis indicated the COG categories as listed on the right of the histogram. The y-axis represented the percentages of the corresponding category among all categories. The number of DEGs belong to the category, and the percentages of the corresponding category among all categories were separately indicated in square brackets. [file 12870_2020_2483_MOESM4_ESM.tif]

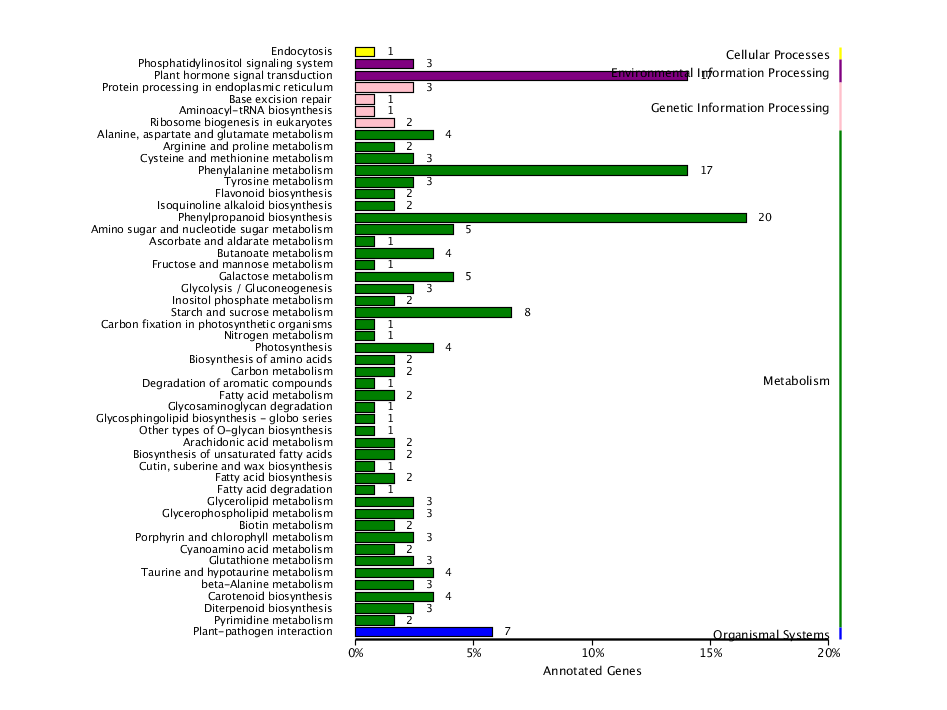

Supplement: Supplementary file 5 — Additional file 5: Figure S5. KEGG classifications of DEGs of foxtail millet in CK14H vs. P14H. The x-axis indicated the percentages of DEGs among the total annotated genes. The left y-axis represented the pathways. The number of DEGs belonged to the annotated pathway were represented after the histograms. [file 12870_2020_2483_MOESM5_ESM.tif]

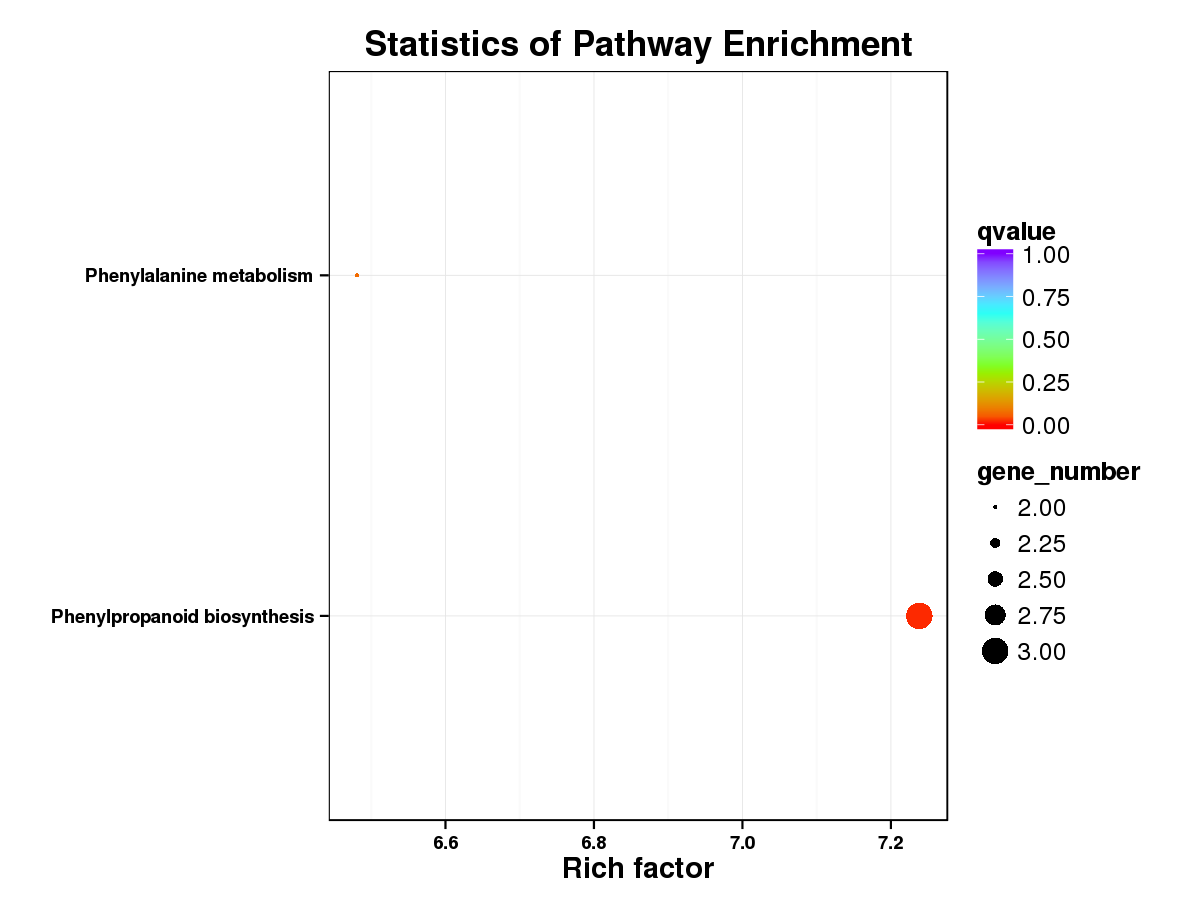

Supplement: Supplementary file 6 — Additional file 6: Figure S6. The pathways with the most significant Q value in CK8H vs. P8H. The x-axis indicated rich factor of DEGs belong to the corresponding pathway. The left y-axis represented the pathways. The sizes of bubble represented the number of DEGs in the corresponding pathway, and the colors of the bubble represented the enrichment Q value of the corresponding pathway. [file 12870_2020_2483_MOESM6_ESM.png]

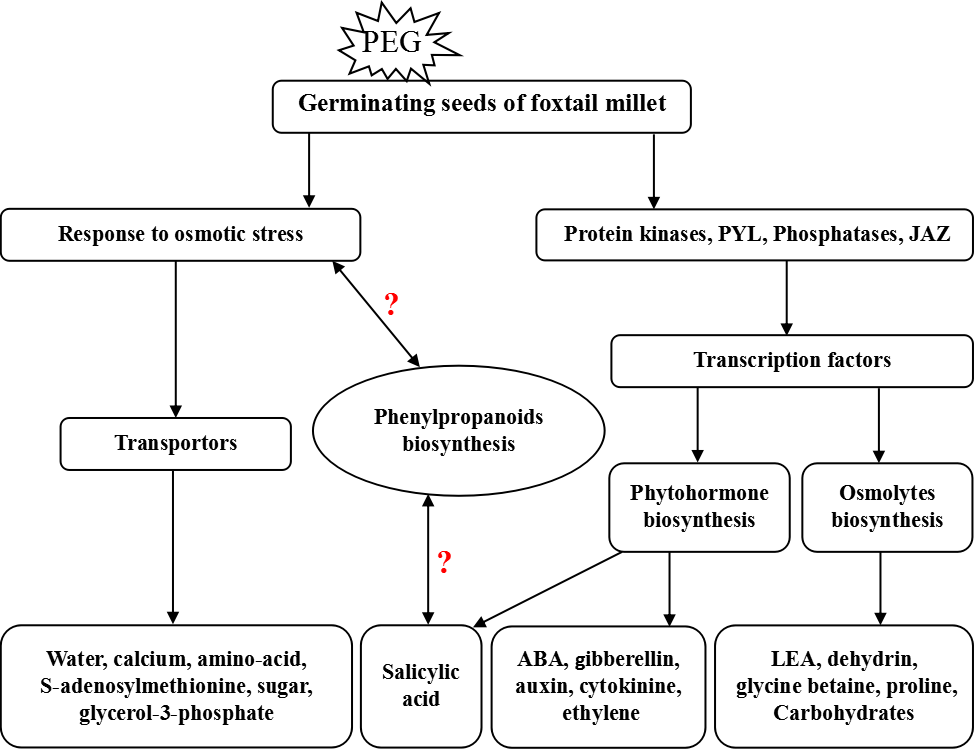

Supplement: Supplementary file 7 — Additional file 7: Figure S7. Schematic representation of PEG stress signal transduction pathway during germination period of foxtail millet. [file 12870_2020_2483_MOESM7_ESM.tif]
