# Supplementary material for: Transcriptome and metabolite analysis reveal the drought tolerance of foxtail millet significantly correlated with phenylpropanoids-related pathways during germination process under PEG stress
Source: BMC Plant Biol. 2020 Jun 15;20:274. doi: 10.1186/s12870-020-02483-4 (PMC7296958; doi:10.1186/s12870-020-02483-4)
Supplement: Supplementary file 8 — Additional file 8: Table S1 - Summary of the sequencing and the reads mapping from the control groups (CK) and the PEG stress groups (P). The sequencing samples were CK2H (germinating for 2 h under normal conditions, then growing for 1 h and 3 h without PEG stress treatment), P2H(germinating for 2 h, then growing for 1 h and 3 h under PEG treatment), CK8H (germinating for 8 h under normal conditions, then growing for 1 h and 3 h without PEG stress treatment), P8H(germinating for 8 h under normal conditions, then growing for 1 h and 3 h under PEG treatment), CK14H(germinating for 14 h under normal conditions, then growing for 1 h and 3 h without PEG stress treatment), P14H (germinating for 14 h under normal conditions, then growing for 1 h and 3 h under PEG treatment). -1 represented the first repeat. -2 represented the second repeat. [file 12870_2020_2483_MOESM8_ESM.docx]

Table S1 Summary of the sequencing and the reads mapping from the control groups ( CK) and the PEG stress groups (P)

|  | CK2H control (phase I) | | P2H stress (phase I) | | CK8H control (phase II) | | P8H stress (phase II) | | CK14H control (phase III) | | P14H stress (phase III) | |
| --- | --- | --- | --- | --- | --- | --- | --- | --- | --- | --- | --- | --- |
|  | CK2H-1 | CK2H-2 | P2H-1 | P2H-2 | CK8H-1 | CK8H-2 | P8H-1 | P8H-2 | CK14H-1 | CK14H-2 | P14H-1 | P14H-2 |
| Clean reads | 38845760 | 39679812 | 35384758 | 36744034 | 56164700 | 43525742 | 52411398 | 47479886 | 46838670 | 49151796 | 41676048 | 48986294 |
| Clean bases | 4894565760 | 4986871932 | 4458479508 | 4617526764 | 7076752200 | 5472201146 | 6603836148 | 5971220182 | 5901672420 | 6178141042 | 5251182048 | 6158961888 |
| GC content | 58.72% | 57.83% | 58.15% | 58.08% | 57.53% | 56.81% | 57.36% | 56.74% | 57.21% | 56.36% | 57.15% | 55.89% |
| Q30 | 89.74% | 85.29% | 89.84% | 85.25% | 90.33% | 85.17% | 89.24% | 85.17% | 91.62% | 85.13% | 91.14% | 85.07% |
| Maped Reads | 31007621 (79.82%) | 31533133 (79.47%) | 29299637 (82.80%) | 28844473 (78.50%) | 45812237 (81.57%) | 35213766 (80.90%) | 42563419 (81.21%) | 38509939 (81.11%) | 38024006 (81.18%) | 39595878 (80.56%) | 33909045 (81.36%) | 39632838 (80.91%) |
| Unique maped reads | 26519605 (68.27%) | 27126368 (68.36%) | 25143769 (71.06%) | 24674729 (67.15%) | 42124995 (75.00%) | 31824703 (73.12%) | 36983205 (70.56%) | 34233419 (72.10%) | 35017461 (74.76%) | 35922812 (73.09%) | 32652815 (78.35%) | 34620597 (70.67%) |
| Multiple maped reads | 4488016 (11.55%) | 4406765 (11.11%) | 4155868 (11.74%) | 4169744 (11.35%) | 3687242 (6.57%) | 3389063 (7.79%) | 5580214 (10.65%) | 4276520 (9.01%) | 3006545 (6.42%) | 3673066 (7.47%) | 1256230 (3.01%) | 5012241 (10.23%) |
